# From disaster to disease: Dengue outbreaks after mining dam failures in Mariana and Brumadinho, Brazil

**DOI:** 10.1016/j.onehlt.2025.101068

**Published:** 2025-05-10

**Authors:** Bianca Alves Almeida Machado, Adivane Terezinha Costa, Paulo de Tarso Amorim Castro, André Talvani

**Affiliations:** a Programa de Pós-Graduação em Saúde e Nutrição, Universidade Federal de Ouro Preto, Ouro Preto, MG, Brazil; b Programa de Pós-Graduação em Evolução Crustal e Recursos Naturais, Universidade Federal de Ouro Preto, Ouro Preto, MG, Brazil; c Escola de Medicina, Universidade Federal de Ouro Preto, Ouro Preto, MG, Brazil; d Programa de Pós-Graduação em Infectologia e Medicina Tropical, Universidade Federal de Minas Gerais, Belo Horizonte, MG, Brazil; e Cátedra UNESCO Água Mulher e Desenvolvimento, Departamento de Geologia, Universidade Federal de Ouro Preto, Ouro Preto, MG, Brazil

**Keywords:** Mining dam, Health, Dengue, Sustainable development goals, Schistosomiasis, One health

## Abstract

The mining dam failures in Mariana (2015) and Brumadinho (2019), Minas Gerais, Brazil, caused catastrophic loss of life and severe environmental damage. The drastic environmental changes caused by mudflows may have contributed to an increase in vector-borne infectious diseases and other public health concerns. This study analyzed infectious disease incidence between 2014 and 2020 using data from the Brazilian DATASUS platform and on-site assessments of rivers and environmental conditions. In Mariana, schistosomiasis notifications remained minimal, while dengue infections rose by over 100 % in both Mariana and Brumadinho. Incidents involving venomous animals remained high but unrelated to mining incidents. Mining collapses appear to have contributed to the rise in dengue and schistosomiasis cases in affected areas. Integrating sustainable development goals into mining practices is crucial to preventing future incidents and the emergence of vector-borne diseases in areas affected by mining activities.

## 1 Introduction

Environmental changes, whether natural or human-induced, drive the global spread of parasites and infectious diseases [1]. The mining dam collapses in Mariana (Nov, 5th, 2015) and Brumadinho (Jan, 25th, 2019), Minas Gerais, Brazil, represent catastrophic disasters caused by corporate negligence. Managers at Samarco Mineração S.A. and Vale S.A. were aware of the impending risks but prioritized profit over human and environmental safety [2]. In this context, the United Nations introduced the *2030 Agenda for Sustainable Development*, emphasizing sustainable development goals (SDG) such as clean water (SDG 6) and health (SDG 3) to prevent such tragedies (sdgs.un.org/goals).

The collapses devastated two key rivers - Gualaxo do Norte in Mariana and Paraopeba in Brumadinho - disrupting ecosystems and community livelihoods [3]. These events forced a breakdown in the natural equilibrium, exacerbating mental health issues and increasing the risk of infectious diseases [4]. Contaminated soil, water, and inadequate sanitation fueled the spread of enteric parasites, waterborne diseases, and vector-borne illnesses such as dengue and schistosomiasis [5,6].

Compounding these challenges, poor medical infrastructure and delays in diagnosis worsened outcomes for affected populations, highlighting failures in SDG 3 [7]. Moreover, climate and environmental changes often facilitate the emergence of mosquito- and tick-borne diseases, as well as other intermediate hosts such as *Biomphalaria* snails, thereby increasing the risk of illnesses like dengue, encephalitis, malaria, and schistosomiasis [8].

This study aimed to identify the most prevalent diseases recorded in the Brazilian government DATASUS platform before and after the collapse of mining dams, evaluate the current health status of rivers affected by the resulting mud and, based on the memory and perception of the local community, assess the extent to which SDGs are currently being implemented.

## 2 Material and methods

### 2.1 Data collection from DATASUS

Health data from the DATASUS platform (https://datasus.saude.gov.br/informacoes-de-saude-tabnet) was accessed through the Ministry of Health to analyze infectious and parasitic diseases reported from 2014 to 2017 (Mariana) and 2018–2020 (Brumadinho). Filters applied included: “Diseases and Injuries of Notification - 2007 onwards (SINAN)” - Accident due to Venomous Animals / Dengue / Schistosomiasis > Minas Gerais > specific years > Municipality of residence (Mariana / Brumadinho).

Additional data from 2023 was evaluated to assess disease notifications and determine whether SDG principles were implemented by mining companies, benefiting community health and river ecosystems. The focus was limited to Mariana and Brumadinho to compare conditions before and after the dam collapses and to gauge public perception of SDG application.

### 2.2 Study population

The study included 80 participants (40 from each region, evenly distributed by gender) who had resided for at least 10 years near the affected rivers: Gualaxo do Norte River (20°22′41″S, 43°25′0″W) and Paraopeba River (20°7′6″S, 44°12′4″W). Participants completed questionnaires in 2023, reflecting on current river conditions and their memories of pre-collapse conditions.

### 2.3 Evaluation of river health

River health was assessed using the “Rapid Bioassessment Protocols” [9,10]. Parameters such as “Bank stability,” “Bank protection by vegetation,” and “Surrounding vegetation conservation” were rated as “very poor,” “fair,” “good,” or “excellent” based on participant scores (very poor: 0–5, fair: 6–10, good: 11–15, excellent: 16–20).

### 2.4 SDG application and perception

To evaluate SDG implementation by mining companies, residents completed an “environmental perception questionnaire” based on [11]. The questionnaire aimed to link residents' perceptions of environmental care with mitigation efforts by the companies responsible for the Fundão (Mariana) and Córrego do Feijão (Brumadinho) dam collapses, particularly regarding the studied SDGs.

### 2.5 Ethical considerations

This study was conducted following the Declaration of Helsinki and approved by the Research Ethics Committee of the Federal University of Ouro Preto (UFOP) -Ethical Assessment CAAE (#47865621.0.0000.5150) and Process #5.060.910. All volunteers signed informed consent forms, approved by the Ethics Committee of the Federal University of Ouro Preto (UFOP). Questionnaires were completed on-site, near both rivers.

## 3 Results

**Fig. 1:**
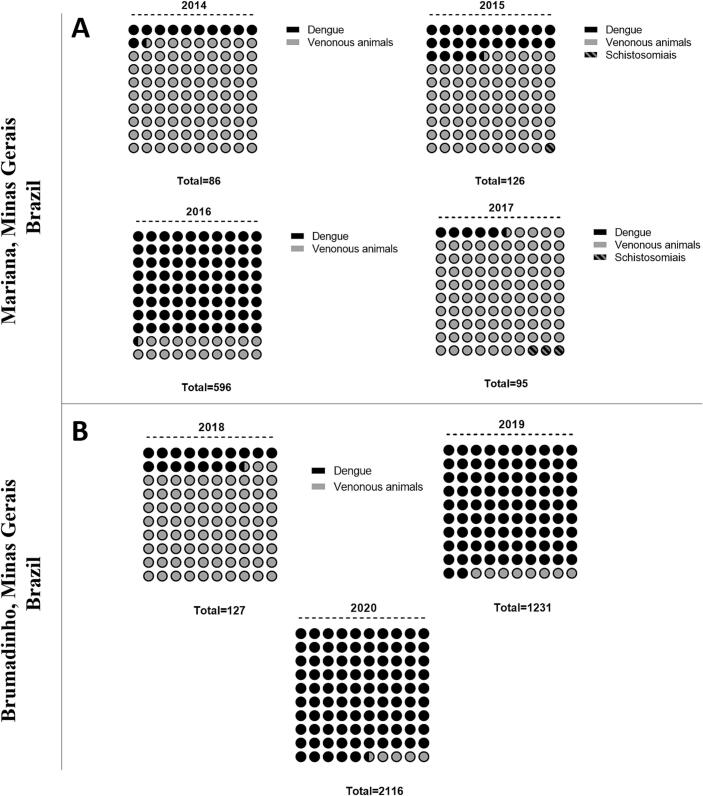
Incidence of dengue, venomous animal accidents, and schistosomiasis in Mariana and Brumadinho, MG, Brazil. This panel represents the incidence of dengue (black circles), venomous animal accidents (grey circles), and schistosomiasis (hatched circles) in Mariana (A) and Brumadinho (B), Minas Gerais, Brazil. Data include notifications for the periods before (2014), during (2015), and after (2016 and 2017) the collapse of the Fundão mining dam in Mariana (A) and the Córrego do Feijão mining dam in Brumadinho (B). The total number of notifications for each year is indicated below the respective scheme.

Following the Mariana mining dam collapse, dengue cases surged in the local population, rising from 10 cases in 2014 to 31 in 2015 and peaking at 479 in 2016, before dropping to 5 in 2017 (Fig. 1A). Schistosomiasis notifications remained minimal, with no cases in 2014 or 2016, 1 in 2015, and 3 in 2017. Accidents involving venomous animals increased by 30 %, from 76 cases in 2014 to 117 in 2016, before decreasing slightly in 2017. No other infectious diseases were recorded during this period. In Brumadinho, dengue cases rose dramatically after the Córrego do Feijão dam collapse, from 22 in 2018 to 1133 in 2019 and 2016 in 2020 (Fig. 1B). No schistosomiasis or other infections were recorded.

**Fig. 2:**
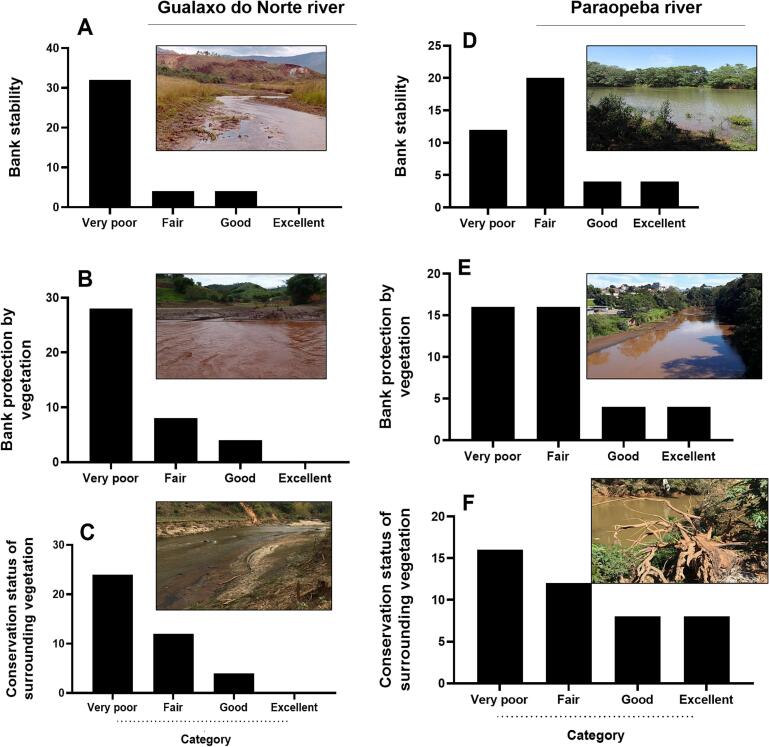
Quality of the Gualaxo do Norte river (left side), affected by Fundão dam rupture, Mariana and, Paraopeba river (right side), affected by Córrego do Feijão mine dam rupture, in Brumadinho. Residents affected by the mining dam collapses in Mariana and Brumadinho assessed the health of the rivers 8 and 4 years after the incidents, respectively. The evaluation covered bank stability (A and D), bank protection by vegetation (B and E), and the conservation status of surrounding vegetation (C and F). These parameters were categorized as very poor, fair, good, or excellent, based on the residents' interpretation of items in the rapid bioassessment protocol and their recollections of river conditions before the collapses. The included images illustrate river health for each analyzed parameter. Image sources: A, C, D, E, F (authors) and B (Dr. Hermínio Nalini – UFOP).

Environmental assessments suggest a possible association between dengue cases and venomous animal incidents, potentially linked to habitat changes caused by mudflows and waste accumulation along riverbanks. Additionally, damage to riverbank protection and the surrounding vegetation, often a result of low conservation efforts (Fig. 2), may also create favorable conditions for the proliferation of vectors and venomous species. Residents reported poor riverbank stability (Fig. 2A and B), degraded vegetation quality (Fig. 2B and E) and conservation of surrounding vegetation (Fig. 2C and F) in 2023 for the Gualaxo do Norte (Mariana) and Paraopeba (Brumadinho) rivers, respectively. In 2023, DATASUS data recorded 2287 dengue cases and 137 venomous animal incidents in Mariana, and 2288 dengue cases, 1 schistosomiasis case, and 284 venomous animal incidents in Brumadinho.

Residents expressed dissatisfaction with post-collapse water and sanitation conditions, with 70–100 % of affected populations reporting dissatisfaction and limited recognition of SDG 6 implementation.

## 4 Discussion

Dengue, a viral disease caused by the *flavivirus dengue virus* and transmitted primarily by *Aedes aegypti* mosquitoes, thrives in diverse habitats, including natural and artificial containers [12]. The ecological plasticity of *Aedes* mosquitoes allows them to adapt to environmental changes, including those caused by the mining dam collapses in Mariana and Brumadinho. These events may have released mining waste, debris, and stagnant water, which, when combined with damaged sanitation infrastructure, could have created favorable conditions for *Ae. aegypti* breeding and possibly driven venomous animals into human habitats. Less than a year later, dengue cases surged. From 2014 to 2016, there were no significant changes in pluviometric indices in Mariana and Brumadinho regions. In Mariana, the key variable was the intensive human activity removing dam debris in 2015, followed by environmental stabilization in 2016, which facilitated mosquito breeding. It is important to note that the intensive search for victims' bodies by local authorities in the months following the tragedy left little opportunity for the collection of mosquito eggs or adults for investigative purposes. However, the impact of the disaster on mosquito populations might still be assessed using traps to collect eggs [13] in areas affected by the mud. However, such measurements must account for both anthropogenic and natural interferences, including those resulting from natural environmental disturbances or human-induced accidents, such as mining dam collapses.

After the dengue outbreak in 2016, municipal authorities implemented strict mosquito control measures, reducing cases in 2017. A similar pattern was observed in Brumadinho. Beyond environmentally favorable, local humidity, temperature, periodic rainfall and high-salinity water [14], other parallel aspects should be considered to gain insight into the increase in dengue cases in affected areas due to mining collapses. Although many studies on rainfall have primarily examined its impact on the distribution of vector populations, they often overlook the direct relationship with human disease incidence. However, mosquito abundance does not necessarily equate to increased pathogen transmission [15]. Other elements of vectorial capacity may significantly influence disease dynamics. For example, the human-biting rate can rise due to accumulated water; environmental changes can increase vector-human contact; and flooding can deteriorate housing conditions, making them more susceptible to vector intrusion. While this study did not mainly focus on vector biology, recognizing the potential timing and strength of causal links, spanning geological, environmental, and biological domains, is essential for improving human-induced or natural disaster response strategies and forecasting disease outbreaks.

Dengue infection can range from mild fever to severe hemorrhagic fever or shock syndrome, influenced by viral factors such as subgenomic RNA and host genetic predispositions [16]. By 2023, reported cases exceeded 2000 in both communities, reflecting the residents' perception of inadequate river health and ineffective Sustainable Development Goal (SDG) implementation.

Environmental changes also contributed to increased accidents involving venomous animals, particularly in Mariana, affecting vulnerable populations [17]. These incidents highlight broader ecological imbalances exacerbated by unplanned urbanization and insufficient environmental management [18]. Conversely, the impact of mining dam collapses on schistosomiasis was minimal, as conditions were unsuitable for the proliferation of *Biomphalaria* snails, the intermediate host of *Schistosoma mansoni* [19]. There were no recorded cases of geohelminth infections (nematode and platyhelminth worms, such as *Taenia*) in the **DATA-SUS** system during the period of the mining dam collapses referenced to Mariana and Brumadinho. However, this does not necessarily indicate the absence of helminth infections but rather potential gaps in monitoring, diagnosis, and reporting. Specific studies and more detailed epidemiological surveillance are needed to assess the true impact of these disasters on helminth transmission, not only in the directly affected areas but also in more distant regions. Notably, rivers and mud carrying potential helminth eggs travelled across multiple municipalities before reaching the Atlantic Ocean in the Espírito Santo State, Brazil.

The lack of measurable application of SDGs by large corporations underscores the urgent need for accountability. Without standardized methods to assess SDG adherence, companies may engage in greenwashing, neglecting meaningful environmental and public health action [20]. Drawing on biological knowledge of dengue fever and other disease control measures, the strict enforcement of the SDGs may help reduce the incidence of these diseases in natural areas adversely affected by industrial and/or mining activities, thereby mitigating associated public health impacts**.**

Residents play a crucial role in the implementation and oversight of SDGs, enabling the scientific community to assess the ongoing impacts of mining dam collapses on both nature and human health. Their active participation also contributes to improving the quality of their newly restructured lives. In addition, rebuilding emotional and economic autonomy is essential to prevent mental health issues and social withdrawal, which often stem from fears of ineligibility for future compensatory benefits. The involvement of the academic community is vital in supporting affected populations across various fields of knowledge, alongside the participation of local leaders in discussions and planning with municipalities, corporations, and governmental authorities. In this sense, Fig. 1 of this manuscript presents a panel of disease cases recorded in the DATA-SUS system before, during, and after the mining dam collapses, while Fig. 2 brings forth the memories and perceptions of the local population regarding the health of the rivers, comparing past and present conditions. Above all, strict and continuous monitoring of financial resources obtained from mining-related fines is imperative. These funds must be allocated transparently and collectively, rather than individually, to ensure their effective application in restoring both the natural environment and the well-being of affected communities.

Regarding public health prepardness in disaster-affected areas, there is crucial the involvement of residents, the Municipal and State Health Departments and the Ministry of Health, discussing planning of actions to mitigate impacts caused by the mining dams collapses and, in parallel, proposing preventive health strategies for potential accident risks in other municipalities (eg. The Casa de Pedra dam, in Congonhas, MG). Finally, we pointed out the importance of expanding the production of scientific knowledge in environmental surveillance and health models, which would support the understanding of mining disasters, given their complexity.

In summary, local populations in Mariana and Brumadinho recognize that the health of river ecosystems, including bank stability, vegetative protection, and the conservation of surrounding areas, was significantly altered by the dam collapse disasters. Supported by data from DATASUS, this study identified an increase in dengue infections and accidents involving venomous animals in both affected regions around one year after the disasters. Grounded in the One Health approach, particularly SDG 6 (Clean Water and Sanitation) and SDG 3 (Good Health and Well-being) into mining operations is essential. Such integration can help mitigate the transmission of vector-borne diseases and other threats to human health, while promoting sustainable development in impacted communities.

## 5 Research limitations and future directions

This study encountered several limitations. Since the mining dam collapsed in both cities, residents have been approached by multiple professionals and researchers. Over time, this has led to resistance and reluctance toward new interviews or interventions. Additionally, many residents fear that sharing their perceptions and experiences could affect their eligibility for compensatory benefits, making them hesitant to participate. There was also limited information concerning specific biological data (vector-borne, geo-helminth-, and other emerging diseases) that may have increased due to the mining dam collapses. If not properly recorded, historical data risks being partially lost. Despite these challenges, fostering the emotional and economic autonomy of the affected population, combined with medical, biological, and environmental research, can help reconstruct the ongoing impacts of the disaster. Survivor memories, when integrated with scientific investigation, serve as a crucial tool for understanding and addressing the long-term consequences of the tragedy.

## CRediT authorship contribution statement

**Bianca Alves Almeida Machado:** Writing – review & editing, Writing – original draft, Resources, Methodology, Investigation, Formal analysis, Data curation, Conceptualization. **Adivane Terezinha Costa:** Writing – review & editing, Writing – original draft, Validation, Supervision, Project administration, Methodology, Conceptualization. **Paulo de Tarso Amorim Castro:** Writing – review & editing, Writing – original draft, Validation, Supervision, Project administration, Formal analysis, Data curation, Conceptualization. **André Talvani:** Writing – review & editing, Writing – original draft, Validation, Supervision, Investigation, Funding acquisition, Formal analysis, Conceptualization.

## Declaration of generative AI and AI-assisted technologies in the writing process

During the preparation of this work, the authors used ChatGPT 3.5 to improve readability and language. After using this tool/service, the authors reviewed and edited the content as needed and take full responsibility for the content of the published article.

## Funding

The author(s) declare that no financial support was received for this research, authorship and, publication of the article.

## Declaration of competing interest

The authors declare this research was conducted without conflicts of interest.

## Data Availability

Data will be made available on request.
